# Incidence and predictors of mortality among adult trauma patients admitted to the intensive care units of comprehensive specialized hospitals in Northwest Ethiopia

**DOI:** 10.1186/s40001-023-01056-z

**Published:** 2023-03-09

**Authors:** Mengistu Abebe Messelu, Ambaye Dejen Tilahun, Zerko Wako Beko, Hussien Endris, Asnake Gashaw Belayneh, Getayeneh Antehunegn Tesema

**Affiliations:** 1 Department of Nursing, College of Medicine and Health Sciences, Debre Markos University, Debre Markos, Ethiopia; 2 Department of Emergency and Critical Care Nursing, School of Nursing, College of Medicine and Health Sciences, University of Gondar, Gondar, Ethiopia; 3 Department of Medical Nursing, School of Nursing, College of Medicine and Health Sciences, University of Gondar, Gondar, Ethiopia; 4 Department of Anesthesia, School of Medicine, College of Medicine and Health Sciences, University of Gondar, Gondar, Ethiopia; 5 Department of Emergency and Critical Care Nursing, College of Medicine and Health Sciences, Bahir Dar University, Bahir Dar, Ethiopia; 6 Department of Epidemiology and Biostatistics, College of Medicine and Health Sciences, University of Gondar, Gondar, Ethiopia

**Keywords:** Incidence, Intensive care unit, Mortality, Trauma patients, Cox regression

## Abstract

**Background:**

Trauma is the leading cause of morbidity and mortality among adult population in the world. Despite many improvements in technology and care, mortality among trauma patients in the intensive care unit is still high particularly in Ethiopia. However, there is limited evidence on the incidence and predictors of mortality among trauma patients in Ethiopia. Therefore, this study aimed to assess the incidence and predictors of mortality among adult trauma patients admitted to intensive care units.

**Methods:**

Institutional-based retrospective follow-up study was conducted from January 9, 2019 to January 8, 2022. A total of 421 samples were chosen using simple random sampling. Data were collected with Kobo toolbox software and exported to STATA version 14.1 software for data analysis. Kaplan–Meier failure curve and log-rank test were fitted to explore the survival difference among groups. After the bivariable and multivariable Cox regression analysis, an Adjusted Hazard Ratio (AHR) with 95% Confidence Intervals (CI) was reported to declare the strength of association and statistical significance, respectively.

**Result:**

The overall incidence rate of mortality was 5.47 per 100 person-day observation with a median survival time of 14 days. Did not get pre-hospital care (AHR = 2.00, 95%CI 1.13, 3.53), Glasgow Coma Scale (GCS) score < 9 (AHR = 3.89, 95%CI 1.67, 9.06), presence of complications (AHR = 3.71, 95%CI 1.29, 10.64), hypothermia at admission (AHR = 2.11, 95%CI 1.13, 3.93) and hypotension at admission (AHR = 1.93, 95%CI 1.01, 3.66) were found significant predictors of mortality among trauma patients.

**Conclusion:**

The incidence rate of mortality among trauma patients in the ICU was high. Did not get pre-hospital care, GCS < 9, presence of complications, hypothermia, and hypotension at admission were significant predictors of mortality. Therefore, healthcare providers should give special attention to trauma patients with low GCS scores, complications, hypotension, and hypothermia and better to strengthen pre-hospital services to reduce the incidence of mortality.

## Background

Trauma has become an international concern due to millions of deaths and disabilities, and it is predicted to be the seventh leading cause of mortality by 2030 in the world [1]. According to World Health Organization (WHO) report, 4.4 million people die every year from unintentional injuries and violence in the world, and it accounts for nearly 8% of overall global mortality [2]. Mortality of trauma specifically due to Road Traffic Accident (RTA) is a growing national concern in Ethiopia [3] and ranks in third place among African nations following South Africa and Nigeria [4]. According to Health Demographic Surveillance (HDS) and Addis Ababa trauma reports about 6.4% and 7% of all deaths are related to trauma, respectively [5, 6], and it is ranked the fourth and fifth leading cause of admission and death, respectively [7].

According to the reviewed literatures, trauma patients accounted for 46.9% of the total Intensive Care Unit (ICU) admitted patients [8], and the mortality of these trauma patients ranged from 3.7 to 28.2% in developed countries [9–13], 23–58% in Africa [14–16] and 12.4–52.8% in Ethiopia [17–19].

Trauma remains the leading cause of death in persons below 45 years of age and it has a negative economic impact due to the loss of socio-economically active people [20]. For each death, there are lots of Emergency Department (ED) visits, ward admissions, ICU admissions, and lifelong disabilities [21]. Currently, it becomes a major challenge in Ethiopia due to increasing accidents, violence, development of technology with poor safety systems, the high population density, and changes in way of living [22].

Trauma patients admitted to the ICU usually presented with injuries in different anatomical areas; head trauma is the most common followed by chest, abdominal, extremity, and pelvic injuries [10, 11, 23, 24]. According to the previous studies conducted across the globe, Glasgow Coma Scale (GCS) score at admission, the severity of the injury, older age, presence of comorbidities, Multi-Organ Failure (MOF), Acute Lung Injury (ALI), sepsis, longer ICU stay, mechanism of injury and traumatic brain injury were independent predictors of mortality among trauma patients admitted in the ICU [10, 13, 14, 25, 26].

Trauma management requires a multidisciplinary approach that begins with pre-hospital care at the trauma site. In the hospital setting, the patients should be stabilized at ED and then admitted to the ICU, which is a multidisciplinary unit struggling with most life-threatening health conditions, where airway support, mechanical ventilation, drug administration and monitoring techniques are provided for patients’ survival [27].

Deaths among trauma patients occur in one of the three phases: immediate death (occurring due to overwhelming injury), early death (occurring within hours after trauma, and delayed death (occurring days to weeks after trauma as a result of treatable infections, MOF or other late complications [1], and during each phase several factors remain associated with the death of trauma patients [28]. Early identification of these factors may have paramount importance for enhancing positive outcomes for a patient with trauma but little is known in low-income countries including Ethiopia [5].

For the past few years many efforts have been done in Ethiopia to reduce the mortality of trauma victims such as purchasing over 3000 ambulances, expansion of emergency care services at health facilities, introduction of diagnostic devices, training human resources, establishment trauma centers [7, 29]. However, the numbers showed that still there are many trauma deaths [7, 17, 18, 29]. Additionally, although trauma is the most common cause of ICU admissions in Ethiopia [30], clinically significant variables such as pre-hospital care and time to death were not investigated by the previous studies. Therefore, this study aimed to assess the incidence and identify predictors of mortality among adult trauma patients admitted to the ICU.

## Methods and materials

### Study design and period

A multi-center institutional-based retrospective follow-up study was conducted among trauma patients admitted to the ICU of four comprehensive specialized hospitals from January 9, 2019 to January 8, 2022.

### Study setting

The study was conducted in comprehensive specialized hospitals in the Amhara region, Northwest Ethiopia. There are five comprehensive specialized hospitals in Northwest Ethiopia; namely the University of Gondar, Tibebe Ghion, Felege Hiwot, Debre Markos, and Debre Tabor comprehensive specialized hospitals. Felege Hiwot and Tibebe Ghion comprehensive specialized hospitals are found in Bahir Dar (the capital city of Amhara regional state) 565 km far from Addis Ababa. Debre Markos and Gondar comprehensive specialized hospitals are found 299 and 730 km far from Addis Ababa, respectively [31]. The first adult ICU in the Amhara region was started in 2009 G.C at Felege Hiwot comprehensive specialized hospital with three beds and two mechanical ventilators. Subsequently, it was expanded to the University of Gondar comprehensive specialized teaching hospital, Debre Markos and Tibebe Ghion comprehensive specialized hospitals in 2011, 2016, and 2019 G.C, respectively. Currently, these hospitals have a total of 44 functional adult ICU beds which has been rendering services for critical patients. The average number of admission to the ICU was 12 patients per month for each hospital, and about 40% of these were trauma patients. The ICUs are providing a similar level of care equipped with mechanical ventilators, noninvasive hemodynamic monitoring devices, portable ultrasounds, defibrillators, and infusion pumps.

### Population

All adult trauma patients who were admitted to the ICU of comprehensive specialized hospitals in Northwest Amhara were the source population. Those trauma patients aged ≥ 15 years admitted to the ICU in selected comprehensive specialized hospitals in Northwest Amhara during the study period were the study population. Those trauma patients whose age was greater or equal to 15 years and had data on the outcome variable were included. However, those whose follow-up time was less than 6 h and trauma patients transferred in from other hospitals’ ICUs were excluded.

### Sample size determination

As there was no similar previous study conducted using the time to an event data model, the sample size was determined by using single population proportion formula with the following assumptions; 52.8% proportion of death among head injury patients in the ICU [17], 95% confidence level and 5% margin of error. Based on this, the actual sample size for the study was computed by using the following formula:

$${\mathbf{n}}\, = \,\frac{{\left( Z \right)2{\text{P}}\,\left( {1 - {\text{p}}} \right)}}{{{\text{d}}^{2} }}\, = \,\frac{{\left( {1.96} \right)2\left( {0.528} \right)\left( {1 - 0.528} \right)}}{{\left( {0.05} \right)2}}\, = \,{\mathbf{383}},$$

where n = sample size, Z = critical value of 95% CI = 1.96, P = Proportion mortality among trauma patients in the ICU (52.8%) = 0.528, D = precision (marginal error) = 0.05.

Thus, by adding 10% for possible incomplete charts and lost medical records, the total sample size was **421.**

### Sampling techniques and procedures

**Fig. 1:**
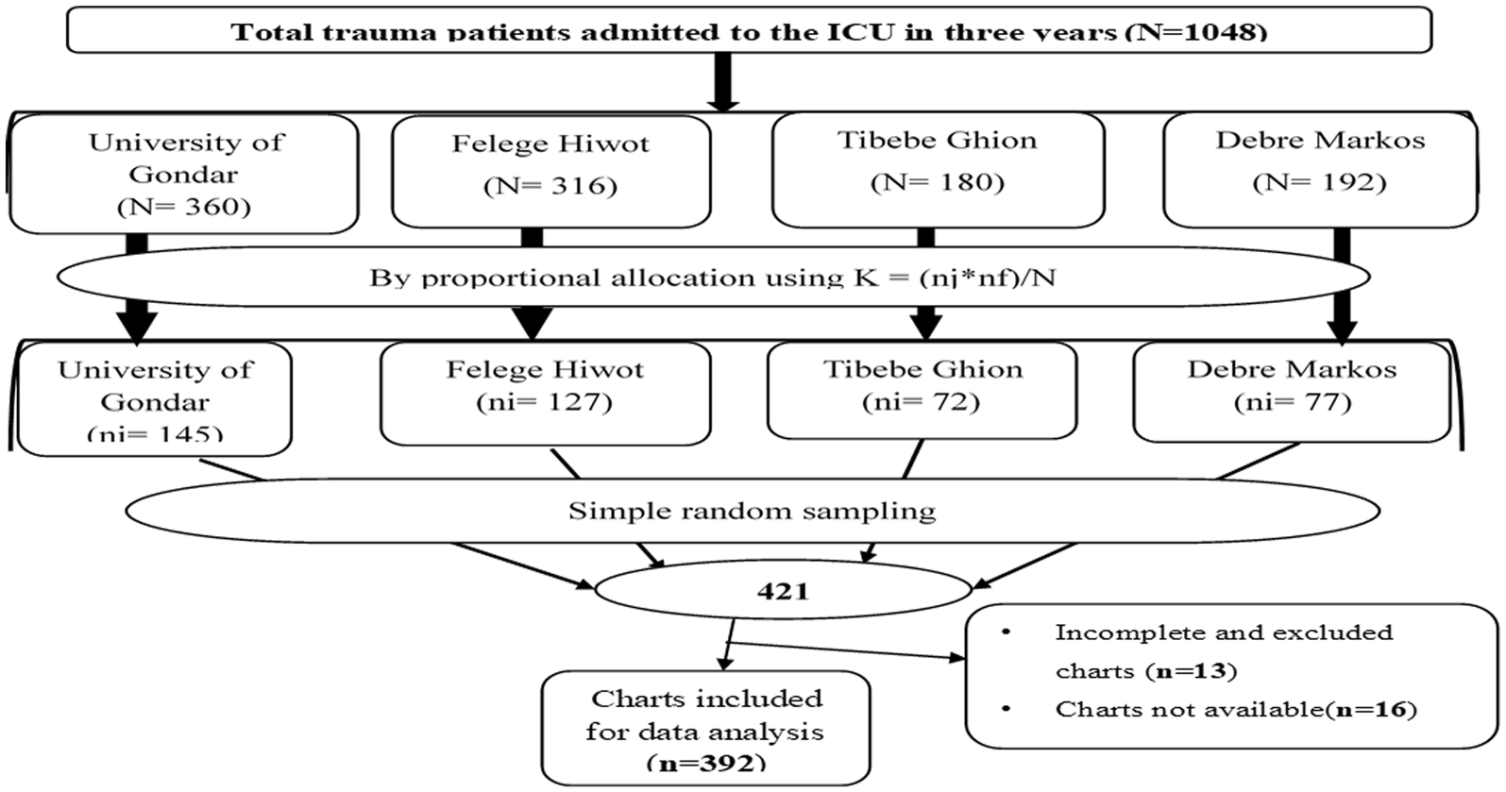
A schematic presentation of sampling procedures used to select trauma patients admitted to the ICU in comprehensive specialized hospitals Northwest Ethiopia, 2022

Four hospitals were included among five comprehensive specialized hospitals in Northwest Amhara. A simple random sampling technique using was employed to select patients’ charts after proportional allocation of the study participants to each hospital. The sampling frame was prepared from a registry of adult trauma patients admitted to the ICU of comprehensive specialized hospitals in Northwest Ethiopia. STATA version 14.1 was used to generate random numbers from the sample frame to select a total of 421 patients’ charts (Fig. 1).

### Operational definitions

#### Censored

Trauma patients who did not develop the outcome of interest (death) like transfer out, left against medical advice, and recovered at the time of ICU discharge.

#### Event

The occurrence of death among trauma patients during the follow-up period.

#### Follow-up time

From the time of ICU admission to the occurrence of an event or ICU discharge in days.

#### Pre-hospital care

Is an emergency care delivered by a professional provider at the scene and during transportation and/or emergency transportation to the health facility by ambulance but outside the walls of the hospital [32, 33].

### Data collection tool and procedures

The English version data extraction checklist was adapted from the different literatures [10, 25, 26, 34] and FMOH triage sheet after being customized according to the variables available in the patient’s chart. Since the record was written in English and data collectors can read and write English, the tool was not translated to the Amharic language. The data were extracted from the registration book and patient charts. The checklist contains socio-demographic data, such as age, sex, and residence; hospitalization-related variables like mode of arrival, pre-hospital care, sources of referral, length of stay; trauma-related data like causes of trauma, mechanism of injury, and area of trauma; clinical data like GCS, Random Blood Sugar (RBS), hemoglobin level, vital signs at admission, complications, comorbidity, interventions in the ICU. All relevant data were collected retrospectively from patient charts by trained 4 BSc ICU nurses using smart phone and tablet-based Kobo collect software with an online server and one MSc nurse was appointed to supervise the overall data collection process.

### Data quality control

A preliminary chart review was done on 22 patient charts at the University of Gondar specialized teaching hospital to ensure the availability of variables on the patient’s chart. The relevance of the variables in the instrument was verified by consulting the expert working in the ICU. Then, appropriate modifications were made based on the inputs before actual data collection. One day of training was given for data collectors and supervisor to ensure consistency and reduce variations between data collectors. Daily communication was made between the principal investigator, supervisor, and data collectors throughout the data collection period. Reviewed charts were boldly marked to avoid re-review. Obtained data were reviewed for accuracy, completeness, clarity, and consistency before analysis.

### Data processing and analysis

The data were exported from an online Kobo collect server to STATA version 14.1 for analysis. Numerical descriptive statistics were expressed by using median with Interquartile Range (IQR) after checking the data distribution with histogram and Shapiro–Wilk test for continuous variables whereas categorical variables were expressed by the frequency with percentage. The outcome of each participant was dichotomized into censored or event. The incomplete data were managed with the assumption of multiple imputations after ascertaining the missing data were completely at random. The Incidence Density Rate (IDR) of mortality was calculated for the entire follow-up period. Kaplan–Meier (KM) failure curve was used to estimate the median survival time and cumulative probability of death and the KM survival curve and the log-rank test were considered to test the presence of difference in the probability of death among the groups. Proportional hazard assumptions were checked both graphically and statistically using log (-log) plot and Schoenfeld residual test, respectively, and it was satisfied (*p* = 0.993). Multicollinearity was checked with variance inflation factor (VIF) and the mean was 1.49 which indicated that there was no significant multicollinearity. In addition, shared frailty was checked to see unobserved heterogeneity between hospitals and the p-value for the likelihood ratio test for theta was non-significant at *p* = 0.375 which showed the classical Cox regression model was the best-fitted model over the Cox frailty model for the sake of model parsimony. The Cox proportional hazard regression was used to explore the association between each independent variable with the outcome variable. The model's fitness was checked by using Cox–Snell residuals test and the hazard function follows 45˚ close to the baseline hazard which indicated that the model was well fitted. For the residual test, it was possible to conclude that the final model fit for data well. Both bivariable and multivariable Cox proportional hazard regression were used to identify predictor variables. Variables having a *p*-value < 0.2 in the bivariable analysis were candidates for the multivariable analysis and Adjusted Hazard Ratio (AHR) with 95% Confidence Intervals (CI) was computed to evaluate the strength of association and variables with a p-a value less than 0.05 were considered as statistically significant with the incidence of mortality among trauma patients.

## Results

### Socio-demographic characteristics of the study participants

A total of 392 patients’ charts were included for the analysis which makes the response rate 93.1%. More than half (58.7%) of the study participants were aged between 15 and 30 years. About 326 (83.2%) of the study participants were males and more than two-thirds (68.7%) were rural residents (Table 1).

**Table 1:** Socio-demographic characteristics of trauma patients admitted to the ICU in comprehensive specialized hospitals of Northwest Ethiopia, 2022

Variables	Categories	Total (N = 392)	Outcome status	
			Death (N = 165)	Censored (N = 227)
Age in years	15–30	230(58.7%)	90(39.1%)	140(60.9%)
	31–45	96(24.5%)	39(40.6%)	57(59.4%)
	46–60	47(12.0%)	23(48.9%)	24(51.1%)
	> 60	19(4.8%)	13(68.4%)	6(31.6%)
Sex	Male	326(83.2%)	137(42.0%)	189(52.0%)
	Female	66(16.8%)	28(42.4%)	38(57.8%)
Residence	Urban	124(31.6%)	50(40.3%)	74(59.7%)
	Rural	268(68.4%)	115(42.9%)	153(57.1%)

Out of the total 392 trauma patients, 178 (45.4%) were transported to the hospital by private taxi followed by ambulance (28.6%). One hundred sixty-one (41.1%) study participants were referred from the hospital and the majority (70.9%) of trauma patients admitted to the ICU did not get pre-hospital care. Two hundred-eighteen (55.7%) were admitted to the ICU due to respiratory problems followed by septic shock (32.1%) (Table 2).

**Table 2:** Hospitalization-related characteristics of trauma patients admitted to the ICU in comprehensive specialized hospitals of Northwest Ethiopia, 2022

Variables	Categories	Total (N = 392)	Outcome status	
			Death(N = 165)	Censored(N = 227)
Mode of arrival	Walking	30(7.7%)	6(20.0%)	24(80.0%)
	With support/carried	72(18.4%)	22(30.6%)	50(69.4%)
	Private car/taxi	178(45.4%)	91(51.1%)	87(48.9%)
	Ambulance	112(28.5%)	46(41.1%)	66(58.9%)
Source of referral	Self	119(30.3%)	46(38.7%)	73(61.3%)
	Health center	86(22.0%)	32(37.2%)	54(62.8%)
	Hospital	161(41.1%)	78(48.4%)	83(51.6%)
	Private clinic	26(6.6%)	9(34.6%)	17(65.4%)
Pre-hospital care	Yes	114(29.1%)	51(44.7%)	63(55.3%)
	No	278(70.9%)	114(41.0%)	164(59%)
Reason for ICU admission	Respiratory problems	218(55.7%)	72(33.0%)	146(67%)
	Septic shock	126(32.1%)	57(45.2%)	69(54.8%)
	Respiratory problem and septic shock	35(8.9%)	33(94.3%)	2(6.3%)
	Others*	13(3.3%)	3(23.1%)	10(76.9%)

Respiratory problem (RF and ARDS), others* (fat embolism, airway obstruction)

*ARDS* acute respiratory distress syndrome, *ICU* intensive care unit, *RF* respiratory failure

Violence was the leading cause of injury (58.9%) followed by road traffic accidents (25.5%). Three-fourths (75.5) of trauma patients sustained penetrating injuries and nearly half (44.7%) of the study participants had isolated head injuries followed by abdominal injuries (15.3%). One hundred eighteen (30.1%) trauma patients had poly-trauma and about 59.2% of the study participants had trauma intentionally. The median time elapsed till medical or surgical care was 7 h (IQR 2–16 h) (Table 3).

**Table 3:** Trauma-related characteristics of trauma patients admitted to the ICU in comprehensive specialized hospitals of Northwest Ethiopia, 2022

Variables	Categories	Total (N = 392)	Outcome status	
			Death (N = 392)	Censored (N = 227)
Cause of injury	RTA	100(25.5%)	46(46.0%)	54(54.0%)
	Fall down accident	51(13.0%)	25(49.0%)	26(51.0%)
	Violence	231(58.9%)	89(38.5%)	142(61.5%)
	Burn	10(2.6%)	5(50.0%	5(50%)
Mechanism of injury	Penetrating	296(75.5%)	123(41.6%)	173(58.4%)
	Blunt	96(24.5%)	42(43.8%)	54(56.2%)
Area of Injury	Head	175(44.7%)	54(30.9%)	121(69.1%)
	Chest	24(6.1%)	5(20.8%)	19(79.2%)
	Abdomen	60(15.3%)	34(56.7%)	26(43.3%)
	Others *	15(3.8%)	5(33.3%)	10(66.7%)
	Poly-trauma	118(30.1%)	68(57.6%)	50(42.4%)
Intention of injury	Unintentional	160(40.8%)	76(47.5%)	84(52.5%)
	Intentional	232(59.2%)	89(38.4%)	143(61.6%)
Time elapsed until care	< 6 h	189(48.2%)	76(40.2%)	113(59.8%)
	6–12 h	78(19.9%)	31(39.7%)	47(60.3%)
	> 12 h	125(31.9%)	58(46.4%)	67(53.6%)

Others * (pelvic injury, extremity injury), violence (intentional injuries such as stab, stick)

*RTA* road traffic accident

About (46.4%) of trauma patients admitted to the ICU had a GCS score < 9 at admission. One in four (25%) and two-thirds (66.3%) of the study participants were hypotensive and had low hemoglobin levels during ICU admission, respectively. Only 13.3% of trauma patients had comorbidity. Major surgery was done for (47.7%) of study participants before ICU admission and 237 (60.5%) trauma patients in the ICU were supported by mechanical ventilation with a median duration of 3 days (IQR 2–5). Nearly two-thirds (64.5%) of trauma patients had a complication and the commonest complication was aspiration pneumonia (36.4%) (Table 4).

**Table 4:** Clinical-related characteristics of trauma patients admitted to the ICU in comprehensive specialized hospitals of Northwest Ethiopia, 2022

Variables	Categories		Total (N = 392)	Outcome status	
				Death (N = 165)	Censored (N = 227)
GCS score at admission	< 9		182(46.4%)	126(69.2%)	56(30.8%)
	9–12		83(21.2%)	21(25.3%)	62(74.7%)
	13–15		127(32.4%)	18(14.2%)	109(85.8%)
Hypoxia at admission	Yes		95(24.2%)	68(71.6%)	27(28.4%)
	No		297(75.8%)	97(32.7%)	200(67.3%)
Hypotension at admission	Yes		98(25%)	70(71.4%)	28(28.6%)
	No		294(75%)	95(32.3%)	199(67.7%)
Hypoglycemia at admission	Yes		17(4.3%)	14(82.4%)	3(7.6%)
	No		375(95.7%)	151(40.3%)	224(59.7%)
Hypothermia at admission	Yes		60(15.3%)	44(73.3%)	16(26.7%)
	No		332(84.7%)	121(36.4%)	211(63.6%)
Hyperthermia at admission	Yes		73(18.6%)	44(60.3%)	29(39.7%)
	No		319(81.4%)	121(37.9%)	198(62.1%)
Low Hgb level	Yes		260(66.3%)	130(50%)	130(50%)
	No		132(33.7%)	35(26.5%)	97(73.5%)
Comorbidity	Yes		52(13.3%)	36(69.2%)	16(30.8%0
	No		340(86.7%)	129(37.9%)	211(62.1%)
Major surgery	Yes		187(47.7%)	84(44.9%)	103(55.1%)
	No		205(52.3%)	81(39.5%)	124(60.5%)
Type of surgery (N = 187)	Laparotomy		72(38.5%)	39(54.2%)	33(45.8%)
	Thoracotomy		10(5.4%)	4(40%)	6(60%)
	Craniotomy		79(42.2%)	32(40.5%)	47(59.5%)
	Fixation and reduction		11(5.9%)	3(27.2%)	8(72.8%)
	Tracheostomy		15(8.0%)	6(40%)	9(60%)
Interventions in the ICU	Enteral nutrition	Yes	376(95.9(%)	156(41.5%)	220(58.5%)
		No	16(4.1%)	9(56.3%)	7(43.7%)
	Vasoactive drugs	Yes	128(32.7%)	50(39.1%)	78(60.9%)
		No	264(67.3%)	115(43.6%)	149(56.4%)
	Blood transfusion	Yes	82(20.9%)	35(42.7%)	47(57.3%)
		No	310(79.1%)	130(41.9%)	180(58.1%)
	MV	Yes	237(60.5%)	130(54.9%)	107(45.1%)
		No	155(39.5%)	35(22.6%)	120(77.4%)
Duration of MV (N = 237)	< 5 days		179(75.5%)	84(48.6%)	95(51.4%)
	5–10 days		31(13.1%)	21(67.7%)	10(32.3%)
	> 10 days		27(11.4%)	25(92.6%)	2(7.4%)
Presence of complication	Yes		253(64.5%)	157(62.1%)	96(37.9%)
	No		139(35.5%)	8(5.8%)	131(94.2%)
Type of complication (N = 253)	Shock		16(6.3%)	13(81.3%)	3(18.7%)
	Infection		23(9.1%)	16(69.6%)	7(30.4%)
	AKI		10(3.9%)	4(40%)	6(60%)
	ARDS		24(9.5%)	12(50%)	12(50%)
	Aspiration pneumonia		92(36.4%)	48(52.2%)	44(47.8%)
	Others*		9(3.6%)	5(55.6%)	4(44.4%)
	Two or more complications		79(31.2%)	59(74.7%)	20(25.3%)

Others*(anemia, electrolyte imbalance, IICP)

*AKI* acute kidney injury, *ARDS* acute respiratory distress syndrome, *GCS* Glasgow Coma Scale, *ICU* intensive care unit, *MV* mechanical ventilation

According to the current study, the proportion of death among trauma patients admitted to the ICU was 42.1% with 95% CI (37.3–47.1), 48.9% of trauma patients were improved and discharged to their homes, and 4.6% and 4.3% were left against medical advice and transferred out, respectively.

**Incidence of mortality among trauma patients**

During the follow-up period, 165 trauma patients admitted to the ICU died making the overall incidence density rate of death 5.47 per 100-day observation (95% CI 4.70, 6.37) and the median survival time was 14 days (95% CI 11, 20). The total follow-up time of this cohort was 3015 days with the median follow-up time of 4 days (IQR of 2–10 days). The minimum and maximum follow-up time was 1 and 48 days, respectively. Among deaths reported, more than three-fourths 128(77.6%) of mortality was observed in the first week after admission to the ICU, and 163(98.8%) of deaths occurred within 30 days of admission. The cumulative probability of death at the end of 1, 10, 14, 20, 30 and 40 days was 11.5%, 43.6%, 50.4%, 57.3%, 68.5%, and 74.8%, respectively.

**Fig. 2:**
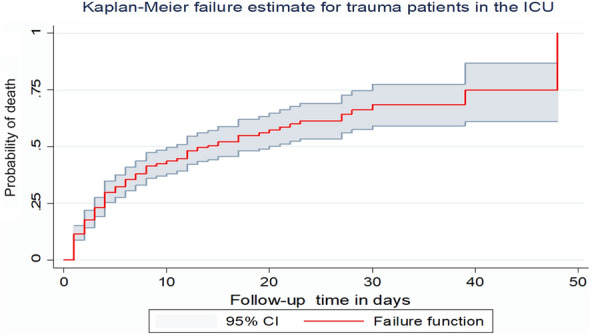
Kaplan–Meier failure curve for trauma patients admitted to the ICU in comprehensive specialized hospitals in Northwest Ethiopia, 2022

A Kaplan–Meier failure curve was used to describe the median survival time and the cumulative probability of death over the follow-up period (Fig. 2).

### Kaplan–Meier curve with log-rank test

**Fig. 3:**
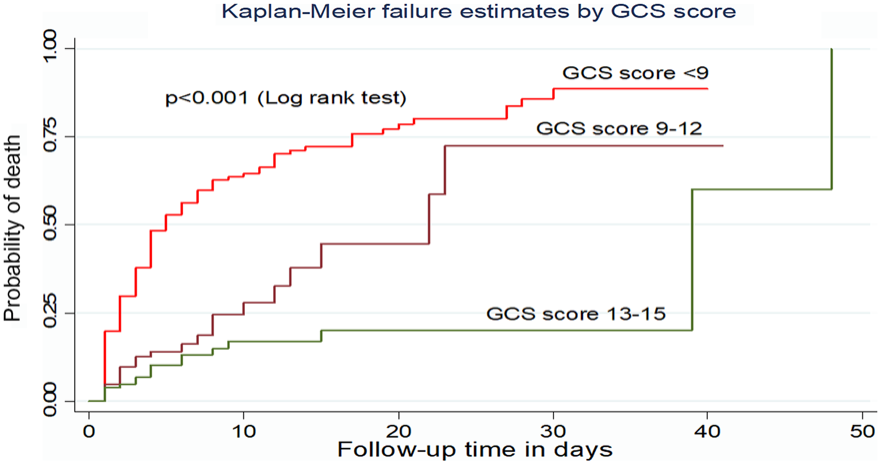
Kaplan–Meier failure curve by GCS score for trauma patients admitted to the ICU comprehensive specialized hospitals in Northwest Ethiopia, 2022

According to the Kaplan–Meier failure curve together with the log-rank test, patients with a GCS score of 13–15 had a longer median survival time (39 days) as compared with those who had a GCS score of  < 9 (5 days). Without adjusting for other covariates, the incidence density rate of death among patients who had GCS scores < 9, 9–12, and 13–15 was 9.8, 3.4, and 1.6 per 100 person-day observation, respectively (Fig. 3).

**Fig. 4:**
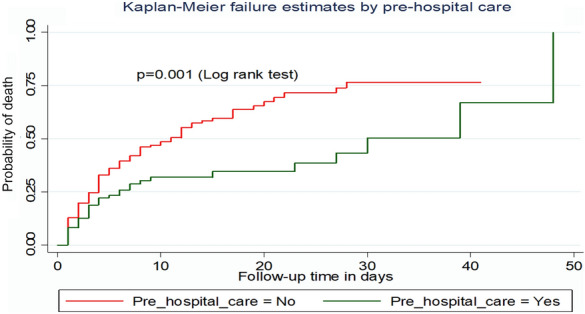
Kaplan–Meier failure curve by pre-hospital care for trauma patients admitted to the ICU comprehensive specialized hospitals in Northwest Ethiopia, 2022

The median survival time for patients who did not get pre-hospital care was 11 days with an incidence density rate of death of 6.2 per 100 person-day observation (Fig. 4).

**Fig. 5:**
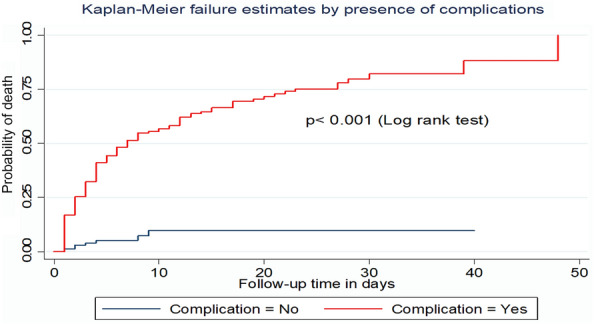
Kaplan–Meier survival curve by the presence of complications for trauma patients admitted to the ICU comprehensive specialized hospitals in Northwest Ethiopia, 2022

Similarly, the incidence density rate of death among patients who had complications was 7.9 per 100-day observations with a median survival time of 7 days (Fig. 5).

**Fig. 6:**
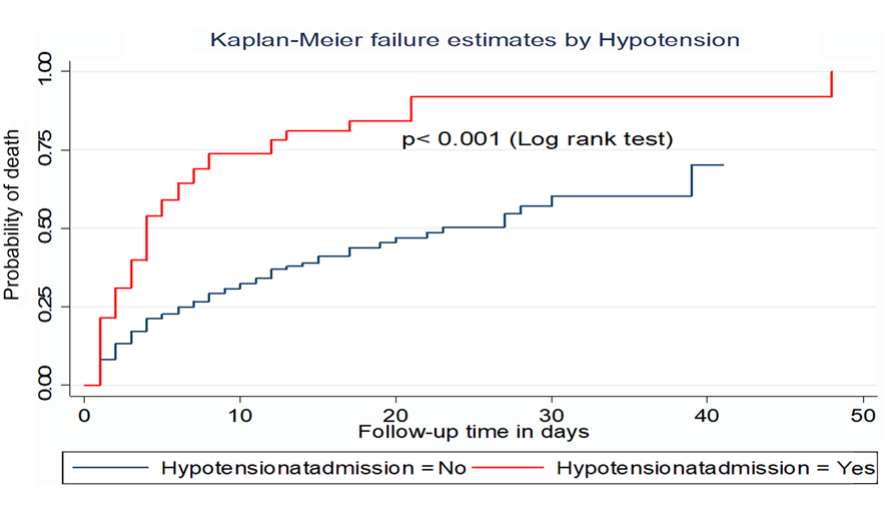
Kaplan–Meier failure curve by hypotension for trauma patients admitted to the ICU comprehensive specialized hospitals in Northwest Ethiopia, 2022

This study also found that the median survival time of patients who were hypotensive at admission was 4 days with IQR (2–12 days) which is shorter compared to those who were not hypotensive at admission (23 days). And, the incidence density of rate of death among patients who were hypotensive at admission was 13.5 per 100-day observation (Fig. 6).

**Fig. 7:**
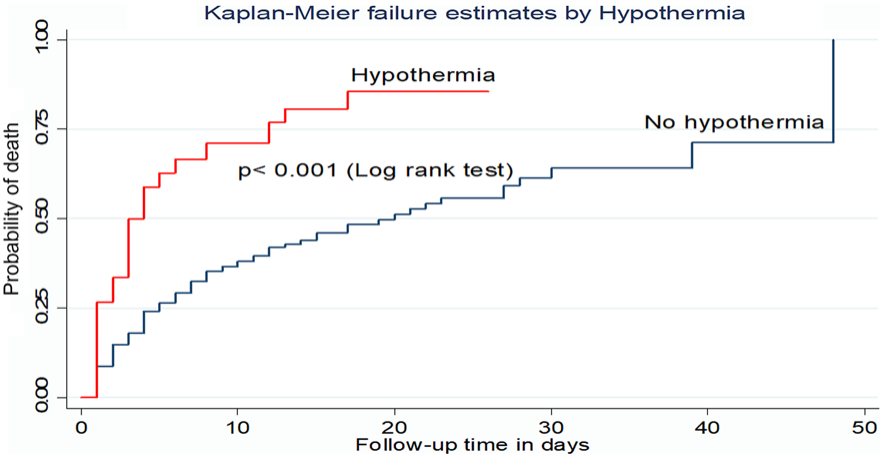
Kaplan–Meier failure curve by hypothermia for trauma patients admitted to the ICU comprehensive specialized hospitals in Northwest Amhara, 2022

According to this study, the incidence density of patients who were hypothermic at admission was 14.1 per 100-day observation with a median survival time of 4 days with IQR (1–12 days) which is lower than their counterparts (20 days) (Fig. 7).

### Predictors of death among trauma patients admitted to the ICU

The risk of death among trauma patients who did not get pre-hospital care was 2 times (AHR = 2.00, 95% CI 1.13, 3.53) higher than those who did get pre-hospital care service. Likewise, the hazard of death among trauma patients who had a GCS score < 9 at admission was 3.9 times (AHR = 3.89; 95% CI 1.67, 9.06) higher compared to those who had a GCS score of 13–15. Trauma patients who had complications were 3.7 times (AHR = 3.71, 95% CI 1.29, 10.64) higher risk of mortality than their counterparts. Furthermore, the risk of death among hypothermic patients at admission was 2.1 times (AHR = 2.11, 95% CI 1.13, 3.93) higher than those who were not hypothermic and similarly, the hazard of death among hypotensive patients at admission was 1.9 times (AHR = 1.93, 95% CI: 1.01, 3.66) higher than those who were not hypotensive (Table 5).

**Table 5:** Bivariable and multivariable Cox proportional hazard regression analysis of predictors of mortality among trauma patients admitted to the ICU in comprehensive specialized hospitals of Northwest Ethiopia, 2022

Variables	Categories	Death	Censored	CHR (95%)CI	AHR (95%) CI
Pre-hospital care	No	129	151	1.84(1.26–2.68)	2.00(1.13–3.53) *
	Yes	36	76	1	1
GCS score at admission	< 9	123	56	5.99(3.61–9.96)	3.89(1.67–9.06) *
	9–12	18	62	2.03(1.07–3.85)	1.44(.53–3.88)
	13–15	24	109	1	1
Hypotension at admission	Yes	70	28	3.75(2.68–5.26)	1.93(1.01–3.66) *
	No	95	199	1	1
Hypothermia at admission	Yes	44	16	2.72(1.92–3.87)	2.11(1.13–3.93) *
	No	121	211	1	1
Presence of complication	Yes	157	96	10.53(5.17–21.44)	3.71(1.29–10.64) *
	No	8	131	1	1

*AHR* adjusted hazard ratio, *AKI* acute kidney injury, *ARDS* acute respiratory distress syndrome, *CHR* crude hazard ratio, *CI* confidence interval, *GCS* Glasgow Coma Scale, *Hgb* hemoglobin, *MV* mechanical ventilation)

^*^p-value < 0.05

## Discussion

According to the current study, the incidence density rate of mortality of trauma patients in the ICU was 5.47 per 100-day observation (95% CI 4.70, 6.37). This finding is higher than the study conducted in United States, 3.3 per 1000-day observation [35]. The reason for this higher figure could be a low number of well-trained staff, inadequate resuscitation drugs, and equipment, level of ICU setup, and the quality of service provided in the ICU.

This study found that the proportion of death among trauma patients admitted to the ICU during the follow-up period is 42.1%. This finding is comparable with the studies conducted in the Republic of Congo (38.8%) [14] and Addis Ababa (46.1%) [18]. However, this finding is higher than the studies conducted in Spain (5.63%) [10], Boston (6.5%) [36], United States (11.2%) [8], Turkey (15.3%, 15.8%) [11, 13], Brazil (22.9%) [24], Greece (27.3%) [25], Brazil 28.2% [26] and Uganda (34.4%) [16]. This high mortality might be due to lack of pre-hospital care, scarcity of ventilators, the difference in the level of ICU setup, and the quality of service provided. Delayed presentation to the health facility may cause this high mortality because in this study nearly one-third of patients took more than 12 h to access medical care. Likewise, this finding is also higher than the study done in Southern Ethiopia which reported that the mortality of trauma patients in the ICU was 12.4% [19]. The possible justification for this difference might be due to the difference in the study population since the study conducted in Southern Ethiopia included all ICU admitted patients and the proportion of trauma patients was low which may account for this lower proportion of mortality.

In contrast, this finding is lower than the studies conducted in Turkey (50%) [37], Nigeria (52.2%) [38], Egypt (58%) [15], Brazil (89.1%) [39], and Ethiopia from Jimma (52.8%) [17]. The reason for this discrepancy might be the difference in the study period, study population, the severity of injury, and duration of follow-up. A study conducted in Ethiopia from Jimma reported that mortality of head injury patients in the ICU and may result in higher mortality.

According to the Cox proportional hazard regression model analysis, did not get pre-hospital care, GCS score less than 9, presence of complication, hypotension, and hypothermia at admission were independent predictors of mortality among trauma patients admitted in the ICU.

This study found that pre-hospital care had a preventive association with the mortality of trauma patients admitted to the ICU. Keeping other variables constant, those trauma patients who did not get pre-hospital care have a double hazard of death as compared to those who did get pre-hospital care. This finding was supported by the studies conducted in Spain [10] and Amsterdam [40]. The evidence suggests that providing pre-hospital care prevents delayed deaths by proper immobilization of unstable fractures, controlling ongoing bleeding, stabilizing vital functions, and rapid transportation by ambulance with trained Emergency Medical Technician (EMT) to an appropriate health facility [41]. However, only 112 (29.1%) of patients had got pre-hospital care in the current study. So, this would imply that strengthening the pre-hospital care system should be one of the major tasks of the responsible body to reduce mortality among trauma patients.

The hazard of death among patients with a GCS score < 9 was 3.9 times higher as compared to those who had a GCS score of 13–15. This finding was supported by studies conducted in Boston [36], Greece [42], Turkey [11, 37], Spain [43], Egypt [15], and Ethiopia [44, 45]. The possible reason could be those patients with lower GCS scores are unable to protect their airways, had a higher risk of aspiration, had compromised ventilator effort, and risk of developing intracranial hypertension which reduces cerebral perfusion and leads to secondary cerebral attacks and death [42]. Maintaining oxygenation and preventing hypercarbia by providing supplemental oxygen and supporting ventilation is critical in managing trauma patients, especially those who have lower GCS scores [1]. Therefore, health care providers should protect their airways and prevent the risk of aspiration by close follow-up and monitoring.

This study showed that the presence of complications such as shock, infection, aspiration pneumonia, AKI, and ARDS had increased the hazard of death by 3.7 times as compared with those who had no complications. This result is supported by the study conducted in Brazil, Greece, Spain, and Turkey [10, 24–26, 42]. The possible reason might be complications that cause multi-organ dysfunction and cellular injury, which in turn may lead to death [26]. For instance, patients with shock usually have hypotension which leads to inadequate tissue perfusion and MOF [1]. Thus, early identification and management of these complications are important for clinical practice before being irreversible.

Hypothermia at admission was also another independent predictor of mortality. The hazard of death among patients who were hypothermic at admission was 2.1 times higher as compared with their counterparts. This finding is consistent with the studies conducted in Netherland and Turkey [46, 47]. This is supported by clinical evidence that cardiac output falls in proportion to the degree of hypothermia and cardiac irritability begins at approximately 33 °C [1]. In addition, hypothermia causes coagulopathy by inhibiting the generation of thrombin and fibrinogen which leads to bleeding and ends in death [48]. Coagulation disorder along with hypothermia and acidosis are referred to as the “trauma triad of death” [49]. Body temperature is an important vital sign to monitor during the management of trauma patients because they are exposed for examinations and may be given room-temperature fluids [1]. Therefore, health care providers should prevent and manage hypothermia by rewarming the patient and environment with appropriate external warming devices, heat lamps, and warmed intravenous fluids.

The current study also found that being hypotensive during ICU admission had a 1.9 times the higher hazard of death when compared to those who were not hypotensive. This finding is supported by the study conducted in Ethiopia [44]. However, a study from Greece showed that hypotension was not significantly associated with the mortality of trauma patients in the ICU [42]. The possible reason might be hypotension reduces vital organ perfusion which leads to multi-organ failures such as renal failure, cardiac failure, and brain death [50]. Therefore, health care providers should be focused on maintaining blood pressure for trauma patients because it is considered as a prerequisite to ensure sufficient tissue perfusion and adequate cerebral blood flow. These measures help to prevent the onset and worsening of cerebral ischemia and life-threatening organ failures.

### Limitations of the study

Due to incomplete data on the ICU logbook and patients’ chart, variables related to physiologic and laboratory parameters necessary to predict mortality such as Acute Physiologic and Chronic Health Evaluation (APACHE) score and injury severity score were not analyzed. In addition, since this study is institutional-based, it could not assess death occurring out of the hospital setting including at the scene and this might underestimate the outcome.

## Conclusion

The overall incidence rate mortality among trauma patients admitted to the ICU was high. Those trauma patients who did not get pre-hospital care, presence of complications, GCS score < 9, hypothermia, and hypotension at admission were independent predictors of mortality which could be minimized by providing appropriate ICU care services. Additional measures have to be undertaken to expand and strengthen the existing pre-hospital care services including ambulance services and it is better to strengthen ICUs and monitor the quality of services in the ICU to decrease the mortality of trauma patients. A prospective cohort study is warranted to overcome the limitations of this study.

## Data Availability

Data will be available upon request to the corresponding author.

## Abbreviations

AHR: Adjusted hazard ratio
AKI: Acute kidney injury
ARDS: Acute respiratory distress syndrome
CHR: Crude hazard ratio
COPD: Chronic obstructive pulmonary diseases
CI: Confidence interval
FMOH: Federal Ministry of Health
GCS: Glasgow Coma Scale
ICU: Intensive care unit
IDR: Incidence density rate
KM: Kaplan–Meier
LOS: Length of stay
MOF: Multi-organ failure
MV: Mechanical ventilation
RBS: Random blood sugar
RTA: Road traffic accident
TBI: Traumatic brain injury
WHO: World Health Organization

## References

[1] ACS (2018). Advanced Trauma Life Support (ATLS) introduction.

[2] WHO. Injuries and violence. 2021.

[3] Ali S, Destaw Z, Misganaw A, Worku A, Negash L, Bekele A (2020). The burden of injuries in Ethiopia from 1990–2017: evidence from the global burden of disease study. Inj Epidemiol.

[4] Aregago G, Gishu T, Getaneh E, Tirore LL, Abame DE, Meskele S (2022). Incidence of mortality and its predictors among patients with head injury admitted to adult intensive care unit at AaBET and ALERT hospitals, Addis Ababa, Ethiopia. J Family Med Primary Care.

[5] Anteneh A, Endris BS (2020). Injury related adult deaths in Addis Ababa, Ethiopia: analysis of data from verbal autopsy. BMC Public Health.

[6] Gelaye KA, Tessema F, Tariku B, Abera SF, Gebru AA, Assefa N (2018). Injury-related gaining momentum as external causes of deaths in Ethiopian health and demographic surveillance sites: evidence from verbal autopsy study. Glob Health Action.

[7] FMOH. Federal Democratic Republic of Ethiopia Four year strategic plan. In: Health, editor. Addis Ababa: FMOH; 2016–2020.

[8] Michetti CP, Fakhry SM, Brasel K, Martin ND, Teicher EJ, Newcomb A (2019). Trauma ICU prevalence project: the diversity of surgical critical care. Trauma Surgery Acute Care Open.

[9] Abe T, Komori A, Shiraishi A, Sugiyama T, Iriyama H, Kainoh T (2020). Trauma complications and in-hospital mortality: failure-to-rescue. Crit Care.

[10] Barea-Mendoza JA, Chico-Fernández M, Quintana-Díaz M, Pérez-Bárcena J, Serviá-Goixart L, Molina-Díaz I (2022). Risk factors associated with mortality in severe chest trauma patients admitted to the ICU. J Clin Med.

[11] Duran M, ULUDAG Ö.  (2020). Mortality analysis of hospitalized trauma patients in the intensive care unit. J Surg Med.

[12] Huei TJ, Mohamad Y, Lip HTC, Noh NM, Alwi RI (2017). Prognostic predictors of early mortality from exsanguination in adult trauma: a Malaysian trauma center experience. Trauma Surg Acute Care Open.

[13] Özlem Ö, Yildiz E, Tokur ME, Gökmen N (2021). Can mortality rate in head and chest trauma patients in the intensive care unit be predicted?. Adnan Menderes Üniversitesi Sağlık Bilimleri Fakültesi Dergisi..

[14] Monkessa CMME, Elombila M, Outsouta GN, Leyono-Mawandza PDG, Ngala MAB-N, Ngono GBTW (2021). Mortality of severe trauma adults patients in Polyvalent Intensive Care Unit at University Hospital of Brazzaville, Republic of Congo. Int J Anesthesia Clin Med.

[15] Beniameen M, Abbas N, Mashhour K, ElGohary T (2017). 32 Predictors of Mortality among head trauma patients reaching ICU. Ann Emerg Med.

[16] Wabule A, Mwanje KA, Obua D, Tumukunde J, Nakibuuka J, Kizito S (2020). Burden, predictors and short-term outcomes of traumatic brain injury among patients admitted to Ugandan intensive care units. Am J Clin Exp Med.

[17] Mosissa D, Alemu S, Rad MH, Yesuf EA. Outcomes of surgical patients admitted to the Intensive Care Unit of Jimma University Medical Center. Health Sci J. 2021:1–4.

[18] Abebe K, Negasa T, Argaw F (2020). Surgical admissions and treatment outcomes at a Tertiary Hospital Intensive Care Unit in Ethiopia: a two-year review. Ethiopian J Health Sci.

[19] Abate SM, Assen S, Yinges M, Basu B (2021). Survival and predictors of mortality among patients admitted to the intensive care units in southern Ethiopia: a multi-center cohort study. Annals of Medicine and Surgery.

[20] Kimura A (2020). International technical transfer of training systems and skills in emergency medicine and trauma management: experiences of the national center for global health and medicine. Japan Global Health Med.

[21] Park Y, Lee GJ, Lee MA, Choi KK, Gwak J, Hyun SY (2021). Major causes of preventable death in Trauma patients. J Korean Soc Traumatol.

[22] FMOH. Trauma management system guideline. Introduction. Addis Ababa: FMOH.

[23] Gün Y, Katırcıoğlu K, Orhon ZN, Gün EÇ, Ayazoğlu TA (2018). Retrospective Analysis of Intensive Care Trauma Patients. Orthopedics.

[24] Pogorzelski GF, Silva TA, Piazza T, Lacerda TM, Netto FAS, Jorge AC (2018). Epidemiology, prognostic factors, and outcome of trauma patients admitted in a Brazilian intensive care unit. Open Access Emerg Med OAEM.

[25] Papadimitriou-Olivgeris M, Panteli E, Koutsileou K, Boulovana M, Zotou A, Marangos M (2021). Predictors of mortality of trauma patients admitted to the ICU: a retrospective observational study☆. Brazilian J Anesthesiol.

[26] Lentsck MH, Oliveira RRD, Corona LP, Mathias TADF (2020). Risk factors for death of trauma patients admitted to an Intensive Care Unit. Rev Lat Am Enfermagem.

[27] Mercer SJ, Kingston EV, Jones CP (2018). The trauma call. BMJ.

[28] Kashani P, Saberinia A (2019). Management of multiple traumas in emergency medicine department: a review. J Family Med Primary Care.

[29] FMOH (2021). Heath Sector Transformation Plan (HSTP) II In Health, editor.

[30] Wondimu S, Bekele S, Giorgis DG, Getachew F, Seyoum N (2018). Pattern of surgical admissions to Tikur Anbessa specialized hospital, Addis Ababa, Ethiopia: a five-year retrospective study. East Central African J Surg.

[31] Yeshaneh A, Tadele B, Dessalew B, Alemayehu M, Wolde A, Adane A (2021). Incidence and predictors of mortality among neonates referred to comprehensive and specialized hospitals in Amhara regional state, North Ethiopia: a prospective follow-up study. Ital J Pediatr.

[32] Sultan M, Zemede B, Zewdie A (2021). Pre-hospital Care to Trauma Patients in Addis Ababa, Ethiopia: hospital-based cross-sectional study. Ethiopian J Health Sci.

[33] Reynolds TA, Calvello EJ, Broccoli MC, Sawe HR, Mould-Millman N-K, Teklu S (2014). AFEM consensus conference 2013 summary: emergency care in Africa-where are we now?. African J Emerg Med.

[34] Kunitake RC, Kornblith LZ, Cohen MJ, Callcut RA (2018). Trauma early mortality prediction tool (TEMPT) for assessing 28-day mortality. Trauma Surg Acute care Open.

[35] Prin M, Li G (2016). Complications and in-hospital mortality in trauma patients treated in intensive care units in the United States, 2013. Inj Epidemiol.

[36] Kisat MT, Latif A, Zogg CK, Haut ER, Zafar SN, Hashmi ZG (2016). Survival outcomes after prolonged intensive care unit length of stay among trauma patients: the evidence for never giving up. Surgery.

[37] Süt N, Memiş D (2010). Intensive care cost and survival analyses of traumatic brain injury. Ulus Travma Acil Cerrahi Derg.

[38] Tobi KU, Azeez A, Agbedia S (2016). Outcome of traumatic brain injury in the intensive care unit: a five-year review. Southern African J Anaesthesia Analgesia.

[39] Quevedo JBIEG, de Lima Escobal AP, de Pinto DM, Silveira NP, Mocellin LP. Clinical, sociodemographic profile and predictors of death in intensive care unit. 2021.

[40] El Mestoui Z, Jalalzadeh H, Giannakopoulos GF, Zuidema WP (2017). Incidence and etiology of mortality in polytrauma patients in a Dutch level I trauma center. Eur J Emerg Med.

[41] Blackwell T. Prehospital care of the adult trauma patient. UpToDate, Waltham, MA. 2011.

[42] Agorogianni D, Michalopoulos E, Prantzou A, Liaskou C, Stamou A, Kapadochos T (2021). Clinical indicators as prognostic factors of multi-trauma patients in the intensive care unit. Health Res J.

[43] González-Robledo J, Martín-González F, Moreno-García M, Sánchez-Barba M, Sánchez-Hernández F (2015). Prognostic factors associated with mortality in patients with severe trauma: from prehospital care to the intensive care unit. Med Intensiva.

[44] Assele DD, Lendado TA, Awato MA, Workie SB, Faltamo WF (2021). Incidence and predictors of mortality among patients with head injury admitted to Hawassa University comprehensive specialized hospital, Southern Ethiopia: a retrospective follow-up study. PLoS ONE.

[45] Amare AT, Tesfaye TD, Ali AS, Woelile TA, Birlie TA, Kebede WM (2021). Survival status and predictors of mortality among traumatic brain injury patients in an Ethiopian hospital: a retrospective cohort study. African J Emerg Med.

[46] Yucel N, Ozturk Demir T, Derya S, Oguzturk H, Bicakcioglu M, Yetkin F (2018). Potential risk factors for in-hospital mortality in patients with moderate-to-severe blunt multiple trauma who survive initial resuscitation. Emerg Med Int.

[47] Balvers K, Van der Horst M, Graumans M, Boer C, Binnekade JM, Goslings JC (2016). Hypothermia as a predictor for mortality in trauma patients at admittance to the intensive care unit. J Emerg Trauma Shock.

[48] Moffatt SE (2013). Hypothermia in trauma. Emerg Med J.

[49] Kander T, Schött U (2019). Effect of hypothermia on haemostasis and bleeding risk: a narrative review. J Int Med Res.

[50] Taghavi S, Askari R. Hypovolemic shock. StatPearls: StatPearls Publishing; 2021. 30020669

